# Assessment of the tumor immune-environment in patients with head and neck cancer

**DOI:** 10.1186/2051-1426-3-S2-P86

**Published:** 2015-11-04

**Authors:** Zipei Feng, Daniel Bethmann, Matthias Kappler, Carmen Ballesteros-Merino, Alexander Eckert, R Bryan Bell, Allen Cheng, Tuan Bui, Rom Leidner, Carlo Bifulco, Claudia Wickenhauser, Bernard A Fox, Barbara Seliger

**Affiliations:** 1 Robert W. Franz Cancer Research Center, Portland, Oregon, USA; 2 Earle A. Chiles Research Institute, Portland, Oregon, USA; 3 Providence Cancer Center, Portland, Oregon, USA; 4 Martin Luther University Halle-Wittenberg, Institute of Pathology, Halle, Germany; 5 Head & Neck Institute, Portland, OR, USA

## Background

Assessment of tumor-infiltrating immune cells using immunohistochemistry (IHC) has been shown to be a powerful way to predict patient prognosis, notably in colon cancer [1, 2]. Similar observations were made in patients with head and neck squamous cell carcinoma (HNSCC), where T cell infiltration is associated with prolonged survival[3,4]. More recently, efforts in multiplex imaging were made to address suppressive mechanisms within the tumor microenvironment and how they impact the overall anti-tumor immune response. We hypothesize that the implications of such work can direct immunotherapy treatment strategies.

## Purpose

Apply multiplex immunohistochemistry of HNSCC samples in order to identify immune biomarkers that correlate with prognosis and to further explore whether relationship analysis provides insights into tumor resistance mechanisms.

## Methods

30 samples from Providence Cancer Center and 80 samples from Halle Medical Center are included in this study. The majority of these samples are HPV negative. Slides were prepared from formalin fixed paraffin embedded (FFPE) samples of patients' primary tumor and stained for CD3, CD8, FoxP3, CD163, PD-L1, Cytokeratin and DAPI using the PerkinElmer Opal system. Digital imaging and analysis were done using PerkinElmer Vectra and inform software. Conventional IHC was performed for analysis of the HLA class I antigen processing machinery (APM) using antibodies directed against HLA class I heavy chain, b2-microglobulin and the transporter associated with antigen processing subunits TAP1 and TAP2.

## Results

Preliminary data from 50 HPV^-^ samples indicated an extremely heterogeneous expression of HLA class I APM components in the HNSCC tumor lesions, which is currently associated with disease progression and immune cell infiltration. In addition, a CD3^+^/CD8^-^/FoxP3^-^ immune infiltrate at the invasive margin was highly predictive of survival (p=0.001). Interestingly, a FoxP3^+^ infiltrate at the invasive margin was also highly predictive of survival (p=0.001). Current studies are evaluating the potential relationships between these two cell populations, as well as their relationship with the tumor immune escape phenotype and with other cells in the tumor environment.

## Conclusion

Preliminary conclusions from our small cohort suggest a strong association between infiltrates of CD3+CD8-cells and increased survival. Interestingly, expression of FoxP3 by these cells did not negatively impact patient prognosis.

## Author's Contribution

ZF and Dr. Bethmann; and Drs. Fox and Seliger contributed equally to this work. Supported by the Harder Family, Lynn and Jack Loacker, Robert W. Franz, Wes and Nancy Lematta, the Providence Medical Foundation and the OMSF (RBB, CBB, BAF).

